# Job Satisfaction, Retirement Attitude and Intended Retirement Age: A Conditional Process Analysis across Workers’ Level of Household Income

**DOI:** 10.3389/fpsyg.2017.00891

**Published:** 2017-05-31

**Authors:** Eleanor M. M. Davies, Beatrice I. J. M. Van der Heijden, Matt Flynn

**Affiliations:** ^1^Huddersfield Business School, University of HuddersfieldHuddersfield, United Kingdom; ^2^Institute for Management Research, Radboud University NijmegenNijmegen, Netherlands; ^3^Department Organization, Faculty of Management, Science and Technology, Open University of the NetherlandsHeerlen, Netherlands; ^4^Kingston Business School, Kingston University LondonLondon, United Kingdom; ^5^Hull University Business School, University of HullHull, United Kingdom

**Keywords:** older workers, intended retirement age, job satisfaction, retirement attitude

## Abstract

In the contemporary workplace, insight into retirement behaviors is of crucial importance. Previous empirical evidence has found mixed results regarding the relationship between work attitudes, such as job satisfaction, and retirement behaviors, suggesting that further scholarly examination incorporating moderating and mediating variables into retirement models is needed. Drawing on comparative models of attitude to retirement, we hypothesized a direct relationship between job satisfaction and intended retirement age for workers with a high household income and an indirect relationship between job satisfaction and intended retirement age, via retirement attitude, for workers with a low or mean household income. We collected data from a sample of 590 United Kingdom workers aged 50+. Using conditional process analysis, we found that the underlying mechanisms in our research model differ according to socio-economic status. We found no direct effect between job satisfaction and intended retirement age. However, an indirect effect was observed between job satisfaction and intended retirement age, via retirement attitude, for both low- and mean-household income individuals. Specifically, the relationship between job satisfaction and retirement attitude differed according to socio-economic group: for high-household income older workers, there was no relationship between job satisfaction and retirement attitude. However, for low- and mean-household income older workers, we observed a negative relationship between job satisfaction and retirement attitude. Otherwise stated, increases in job satisfaction for mean and low household income workers are likely to make the prospect of retirement less attractive. Therefore, we argue that utmost care must be taken around the conditions under which lower income employees will continue their work when getting older in order to protect their sustainable employability.

## Introduction

Populations ages are rising in the United Kingdom and more broadly the developed world, and alongside demographic changes, retirement ages are also increasing. United Kingdom retirement ages have risen 1.2 years for men and 1.4 years for women, respectively, since 2004. At the same time, the government is raising State Pension Ages, with current policy projected to reach 70 in 30 years (Cridland, 2016). Given the actual and normative pressures to extend working life and because it is potentially amenable to intervention by employers (Kautonen et al., 2012), there have been calls for research to give insight into the motivational factors impacting retirement plans (Taylor et al., 2016). As the most significant transition in later adulthood, retirement provides an opportunity for workers to re-evaluate their roles and identity, and requires the development of non-work based activities (Reitzes et al., 1996). Retirement intentions have been the focus of extensive scholarly research in recent years and, in addition to demographic and personal factors such as financial position (Beehr et al., 2000), gender (Feldman, 1994; Talaga and Beehr, 1995; Quick and Moen, 1998), marital status (Feldman, 1994; Szinovacz, 2003), health (Topa et al., 2009) and age (Beehr, 1986; Taylor and Shore, 1995), more recently, scholars have also examined psychological factors affecting retirement including job satisfaction (Mein et al., 2000; Adams et al., 2002; Fisher and Herrick, 2002; Sibbald et al., 2003; Dendinger et al., 2005; Davies and Cartwright, 2011; Kautonen et al., 2012; Oakman and Wells, 2013), organizational commitment (Adams, 1999; Schmidt and Lee, 2008), job-related stress (Wahrendorf et al., 2013), work-family conflict (Raymo and Sweeney, 2006), job demands and control (Blekesaune and Solem, 2005; Elovainio et al., 2005; Harkonmäki et al., 2006; Oakman and Wells, 2013), social networks and cohesion (Henkens and Tazelaar, 1997; Mein et al., 2000; Kosloski et al., 2001; Oakman and Wells, 2013), retirement self-efficacy (Taylor and Shore, 1995; Van Solinge and Henkens, 2005; Topa and Alcover, 2015), and older worker’s identity (Zaniboni et al., 2010; Bayl-Smith and Griffin, 2014; Topa and Alcover, 2015).

As giving up work as a dominant life sphere is a key feature of retirement (Newman et al., 2012), job satisfaction has been considered to be an important factor during retirement decisions (Kosloski et al., 2001). As a central work-related construct, the relationship between job satisfaction and retirement remains a core focus of interest to scholars because individuals’ evaluations, beliefs and feelings about both their job and the idea of leaving their job is likely to influence their retirement behaviors. Prior studies have yielded inconsistent results suggesting that further scholarly examination incorporating moderating and mediating variables into retirement models is needed to advance our understanding (Bidewell et al., 2006; Aguinis et al., 2011).

**FIGURE 1:**
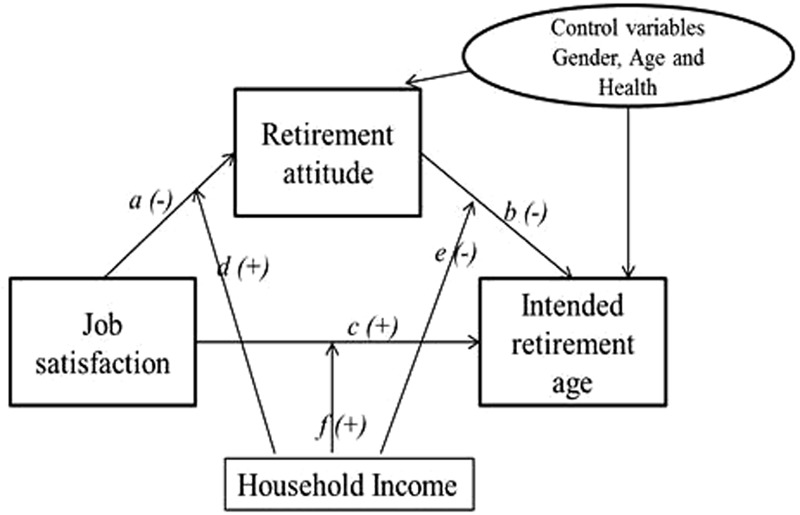
Research model.

In this paper, we investigate whether the way workers anticipate their future state of retirement (retirement attitude) mediates the relationship between job satisfaction and intended retirement age at different levels of household income (see **Figure 1**). Our focus is on the *intended* retirement age of employees 50+ who are still in work rather than the *actual* retirement age of those who have already permanently left the labor market. We do so for two reasons. First, from a theoretical perspective, we are interested in the relationship between present job satisfaction and retirement planning. A focus on actual retirement age would necessitate a retrospective approach to how retirees had felt about their jobs, with substantial hindsight, and thus weaken the link between the two. Second, from a practical perspective, employers are interested in whether and how job satisfaction influences current retirement plans. This study contributes to the prior retirement literature by assessing, first, the robustness of job satisfaction as a predictor of intended retirement age, second, by investigating the possible mediating role of retirement attitude in this relationship, and, third, by examining the parameters of the relationship when socio-economic status (household income) is taken into consideration. As we have noted above, several demographic and personal factors have been shown to influence retirement age. We explicitly focus on socio-economic status as it has been identified as a significant public policy concern within the context of rising pension ages. Specifically, old age poverty and the limited employment choices for older low-skilled workers necessitate a better understanding of the impact of class and income on retirement patterns (Lain, 2012).

### Job Satisfaction and Intended Retirement Age

Job satisfaction is an attitude, defined by Weiss (2002, p. 175) as “*a positive (or negative) evaluative judgment one makes about one’s job or job situation”* which incorporates overall evaluative judgments about a job, affective experiences at work, and beliefs about a job. Both the turnover literature (March and Simon, 1958; Mobley et al., 1979; Holtom et al., 2008; Hayes et al., 2012) and the retirement literature (Mein et al., 2000; Topa et al., 2009; Smith et al., 2011; Kautonen et al., 2012) inform the theorized relationship between job satisfaction and intended retirement age. High job satisfaction is assumed to be a valuable psychological resource which influences the desirability of movement from the organization (March and Simon, 1958) and which an employee is unlikely to wish to relinquish through pronounced earlier retirement. This mechanism is consistent with the notion of continuity in relation to retirement (Atchley, 1989) which assumes that older individuals seek to preserve their existing internal and external continuity when making life choices. So, it is assumed that more highly satisfied employees tend to place higher value on their work (Cytrynbaum and Crites, 1989), are likely to experience greater discontinuity at the onset of retirement, and so will seek to prolong employment and delay retirement. By contrast, older workers who perceive their job negatively, who experience lower satisfaction, and who derive little psychological satisfaction from it, are likely to seek ways to minimize the negative feelings associated with their job by retiring earlier rather than later.

Empirical evidence, however, has not yielded consistent support for the hypothesized association between job satisfaction and intended retirement age. Although some scholarly work supported the expected relationship (Hanisch and Hulin, 1990; Mein et al., 2000; Smith et al., 2011; Kautonen et al., 2012) others did not (McCune and Schmitt, 1981; Taylor and Shore, 1995; Adams and Beehr, 1998; Adams, 1999; Beehr et al., 2000; Davies and Cartwright, 2011; Smith et al., 2011; Post et al., 2013). Some studies have offered more nuanced perspectives. Bidewell et al. (2006), for example, distinguished between intrinsic and extrinsic job satisfaction and found that extrinsic job satisfaction was significantly associated with later increased retirement age whereas intrinsic job satisfaction appeared to be unrelated to preferred retirement age. In a similar vein, Kalokerinos et al. (2015) found that job satisfaction was negatively associated with phased retirement (a form of diminishing employment over time) which is consistent with the preference for continuity for highly satisfied employees.

All in all, the inconclusive empirical results from previous studies suggest that the relationship between job satisfaction and intended retirement age is not straightforward and that more needs to be known about the underlying mechanisms. In this particular contribution, we focus specifically on retirement attitude as a possible mediator in the relationship between job satisfaction and intended retirement age, and we will incorporate the possible moderation effect of household income.

## Mediating Effect of Retirement Attitude

Retirement attitude can variously refer to attitudes toward *retirement* (the role state of being retired), the act of *retiring*, and to attitude toward retirees (see Newman et al., 2012). We focus on retirement attitude as a valenced generalized cognitive evaluation of the expected state of retirement (Hanisch and Hulin, 1991; Post et al., 2013). This form of retirement attitude is usually a progressive transition, in which an anticipatory attitude is formed in the pre-retirement period before any actual ‘event’ takes place (Atchley, 1976; Pinquart and Schindler, 2007). Anson et al. (1989) argued that workers normally engage in a process of anticipatory socialization as they approach retirement (Merton, 1958), and that they adjust their attitude and expectation toward their own retirement in view of the approaching event. Glasmer (1981) suggested that during the latter years of one’s working life, employees cognitively adjust their views on the importance of work so that they arrive at a position of cognitive balance by the time that retirement occurs (p. 106), thereby avoiding cognitive dissonance (Festinger, 1962). Some employers offer ‘phased retirement’ work arrangements in order to facilitate such planned adjustments out of work (Urwin et al., 2013).

As a life stage, retirement usually signals the end of work as a dominant life sphere and, unlike other forms of quitting a job, in retirement, once a person stops paid-work, it is typically not substituted by similar activities (Newman et al., 2012). Retirement characteristically entails multiple life changes and is closely interlinked to other social structures, such as family relationships, social relationships, professional identity, financial position (Szinovacz, 2003) as well as to changes in the organizing of one’s daily life (Pinquart and Schindler, 2007; Wang and Shultz, 2010). Given the potentially profound nature of these changes in the life course, retirement attitude can vary significantly between individuals. Hornstein and Wapner (1985) captured this affective flavor of retirement in their four categories of retirement: (1) ‘retirement as a new beginning’; (2) ‘retirement as beginning of the end’; (3) ‘retirement as a continuation’; and (4) ‘retirement as imposed disruption.’

Empirically, strong associations have been found between the predictive ability of broad, positive retirement attitude and intended retirement age: people who expect to enjoy retirement are more likely to retire earlier than those who expect to be bored in retirement (Feldman, 1994; Hansson et al., 1997; Bidewell et al., 2006) and, indeed, positive expectations of retirement have been associated with lower intended retirement age (Zappala et al., 2008; Davies and Cartwright, 2011; Cochran et al., 2012).

It might be expected that attitude to work and attitude to retirement are inversely related to one another such that a worker with high job satisfaction might be expected to have a more negative attitude to retirement because the act of retiring requires foregoing a source of positive psychological well-being such as one’s passion for work (Houlfort et al., 2015), socio-economic status, income (Post et al., 2013), maintaining lifestyle (Atchley, 1976) and keeping active (Illmarinen et al., 1997). Likewise, a person with lower job satisfaction might be expected to have a positive attitude to retirement because retirement sanctions the cessation of an unrewarding job, implies freedom from the pressures and demands of work, eliminates a source of stress, imposed time constraints, difficult political environments, and so on.

The nature of the relationship between job and retirement attitude has been framed as a comparative process (Anson et al., 1989; Newman et al., 2012; Chevalier et al., 2013) in which the state of work is compared to the state of retirement. Anson et al. (1989) considered retirement attitude in terms of the overall assessment of the *gains* and *losses* associated with both ‘leaving work’ and ‘entering retirement.’ Analogously, Newman et al. (2012) proposed that retirement attitude is formed from the sum of comparing *what is given up in retirement* against *what is gained in retirement*. Chevalier et al. (2013) referred to push (negative work-related factors that push an older worker into retirement), pull (positive perceptions that pull an older worker into retirement), anti-push (feelings of attachment to the current job) and anti-pull (costs and risks associated with retirement) factors to capture the complex influences on retirement decisions. As governments, especially in Europe, are shifting welfare states toward encouraging longer working lives, the academic focus has expanded to include need factors (delaying retirement for financial reasons) and stay factors (being encouraged to delay retirement by positive work environments) in empirical models in this knowledge domain (Ebbinghaus and Hofäcker, 2013). Although the theoretical frameworks that have been outlined above illustrate the central role of attitude to the job in forming attitude to retirement, they also highlight the importance of non-work factors. For instance, retirement may provide gains such as increased free time, and the opportunity to pursue other hobbies, to develop new roles, to undertake voluntary and civic work, to devote time to one’s family and friends, and to access welfare benefits, which are outcomes that may be quite independent of an individual’s attitude to his/her job.

There is some scholarly debate regarding whether the gains made in one domain may compensate for the losses in another domain. Does, for example, a gain in increased family time, compensate for the loss of job-related status? Anson et al. (1989) argued that gains accrued in the non-work domain are unlikely to affect the overall perception of the gains and losses associated with leaving work because although leisure activities may serve as a substitute for work-related activities, the *‘void is still there and the work-related losses do not necessarily fade away*’ (Anson et al., 1989, p. 189). Or, retirement might provide relief from the burden of work, but it does not necessarily follow that work will be replaced by more satisfying activities.

The approach suggested by these comparative frameworks by Anson et al. (1989) and Newman et al. (2012) predicts that individuals will develop a broad-based overall attitude to retirement, based on their evaluation of the expected balance between the gains and losses associated with leaving working and being retired, and that is shaped in part by the expected disruption to their lifestyle (Pearlin et al., 1981; Kessler et al., 1985; Szinovacz, 2003; Burke, 2006). The net balance of perceived gains and losses will vary between individuals, with some older adults expecting greater gains or losses than others (Pinquart and Schindler, 2007). Moreover, given the scale and scope of potential changes across multiple life domains, attitude to retirement is likely to be characterized by attitudinal ambivalence in which individuals will hold both favorable and unfavorable attitudes toward the object of retirement simultaneously (Kaplan, 1972; Newman et al., 2012; Muratore and Earl, 2015). In sum, the discussion above leads us to suggest that job satisfaction and attitude to retirement are related but distinct constructs. In light of the unclear empirical findings, we therefore advance that different pathways operate between job satisfaction, retirement attitude and intended retirement age, for instance, depending on socio-economic status.

## Conditional Effect of Socio-Economic Status

The relationship between retirement and its antecedents is known to vary between social groups (Szinovacz, 2003) and, in this study, we focus specifically on socio-economic status (measured by household income) as a potential moderator. Household rather than individual income has been shown to be the dominant influence over retirement decisions as this encompasses family resources which are available to finance retirement (Loretto and Vickerstaff, 2013). Economic approaches to retirement age suggest that, when they have a choice, workers will retire at the point when they assess that their accumulated financial resources (considering future economic conditions) allow them to support themselves in retirement (Quinn and Burkhauser, 1990; Guillemard and Rein, 1993; Hatcher, 2003; Wang and Shultz, 2010). Correspondingly, lower household income workers, having had less opportunity to accumulate sufficient financial resources over their lifetime, are less likely to be able to exit the workforce through early retirement (Mein et al., 2000) and may need to work longer to maintain their lifestyle than those on higher income (Post et al., 2013). Moreover, they are also less likely to have engaged in formal and informal financial planning (Taylor and Geldhauser, 2007). Many earlier scholarly studies have supported this line of reasoning, finding that a higher financial status is indeed associated with earlier retirement (Flippen and Tienda, 2000; Kim and Feldman, 2000; De Wind et al., 2015).

Although, previously, researchers have noted that relationships between retirement and its antecedents are likely to vary by occupational status, just a few studies have explicitly examined differences in the relationships between job satisfaction, retirement attitude and intended retirement age for different categories of workers. In comparing self-employed and salaried earners in Finland, Kautonen et al. (2012) found that job satisfaction was only a significant determinant of the intended retirement age of individuals who were less satisfied with other life domains, suggesting that satisfaction with other life domains does influence the relationship between job satisfaction and intended retirement age as well. “*A likely interpretation is that for those who are highly satisfied with their leisure time and family life, these domains of life form salient considerations in the retirement decision while the inherent aspects of the work domain, captured in job satisfaction, are a less relevant concern*” (Kautonen et al., 2012, p. 436).

## Hypotheses

Human capital theory (Becker, 1975) predicts that individuals with a higher household income will have stronger financial resources (such as life savings or pension benefits), be more highly skilled, occupy higher status jobs, and so enjoy greater autonomy and control. They are also likely to enjoy superior resources such as increased social capital, professional and non-work networks (e.g., civic roles and leisure opportunities) which are invaluable as well in easing the retirement transition (Muratore and Earl, 2015). As such, from this privileged position, higher socio-economic groups will be freer to respond more directly to their positive or negative evaluation of their job. Therefore, for higher household income workers, we hypothesize that the decision-making around intended retirement age will be relatively less complex, and for a direct relationship to be found between job satisfaction and intended retirement age. Lower socio-economic groups, on the contrary, will have less freedom to respond to a positive or negative evaluation of their job. Having accumulated fewer financial and social resources to draw upon, they face greater risk in the retirement transition, and, as a result, their retirement decision will therefore be more complex. Instead of responding directly to their positive or negative job attitude, they will need to engage in a more complex psychological process of comparing the state of work and the state of retirement.

We therefore hypothesized that the pathways between job satisfaction, retirement attitude and intended retirement age will be moderated by household income, and have formulated the following:

Hypothesis 1: There will be a direct relationship between job satisfaction and intended retirement age for workers with a high household income, but not for those with a low or mean household income.
Hypothesis 2: There will be an indirect relationship between job satisfaction and intended retirement age, via retirement attitude for workers with a low or mean household income, but not for those with a high household income.

## Methodology

### Sample and Procedure

Data were obtained through telephone interviews among a sample of 800 people in work over the age of 45 from the United Kingdom. There is no standard definition of ‘older worker,’ but 50 years or older one is frequently used in scholarly studies to denote older workers (Ekerdt, 1998; Zaniboni, 2015). In line with this approach, 50 years or older was selected for this analysis, yielding a sample of 670. There were 80 non-responses to the question on intended retirement age. To test for possible differences between respondents and non-respondents to the intended retirement age question, the samples were compared on key demographic variables using chi-square test for categorical variables and independent sample analysis of variance for ordinal variables. The demographic characteristics for response and non-response samples are shown in **Table 1**. These procedures did not reveal any evidence that intended retirement age responses were not missing at random and so subsequent analysis was conducted on the resulting sample of 590 responses. The mean age of the included respondents was 57.32 years (*SD* = 4.39), their mean intended retirement age was 65.18 years (*SD* = 4.39), and 50% of the sample was male. Data were collected across a broad range of industry sectors.

**Table 1:** Sample demographic characteristics for sample responding to intended retirement age question (*n* = 590) and non-respondents to intended retirement age question (*n* = 80).

		Respondents		Non-respondents	
		Frequency (*n* = 590)	Percent	Frequency (*n* = 80)	Percent
Gender	Male	294	0.50	39	0.49
	Female	296	0.50	41	0.51
Sector	Agriculture	2	0.00	0	0.00
	Energy and water	15	0.03	2	0.03
	Manufacturing	46	0.08	4	0.05
	Construction	25	0.04	4	0.05
	Catering (e.g., hotel or restaurant)	13	0.02	0	0.00
	Transport	42	0.07	5	0.06
	Banking and finance	29	0.05	2	0.03
	Public administration	27	0.05	1	0.01
	Education	55	0.09	6	0.08
	Health services	61	0.10	2	0.03
	Charity/voluntary sector	25	0.04	5	0.06
	Retail and wholesale	52	0.09	7	0.09
	Social care and social work	22	0.04	4	0.05
	Business and support services	55	0.09	10	0.13
	Others	121	0.21	28	0.35
Marital Status	Single (never been married or cohabiting)	62	0.11	16	0.20
	Married or cohabiting	411	0.70	46	0.58
	Divorced	99	0.17	17	0.21
	Widowed	18	0.03	1	0.01
Region	East Midlands	37	0.06	6	0.08
	Eastern	41	0.07	6	0.08
	London	55	0.09	11	0.14
	North	37	0.06	4	0.05
	North West	66	0.11	8	0.10
	Northern Ireland	12	0.02	1	0.01
	Scotland	65	0.11	8	0.10
	South East	101	0.17	16	0.20
	South West	50	0.08	6	0.08
	Wales	20	0.03	5	0.06
	West Midlands	56	0.09	3	0.04
	Yorkshire and Humber	50	0.08	6	0.08
Weekly household income	Below £237 per week (Bottom 20% of United Kingdom households)	57	0.10	14	0.18
	Between £238 and £412 per week (20–39%)	176	0.30	28	0.35
	Between £413 and £650 per week (40–59%)	187	0.32	21	0.26
	Between £651 and £1014 per week (60–79%)	119	0.20	10	0.13
	Over £1014 per week (The top 20% of United Kingdom households)	51	0.09	7	0.09
Education level	Higher degree (e.g., Masters or Ph.D.)	46	0.08	6	0.08
	First degree (e.g., BA, BSc)	109	0.18	15	0.19
	Other qualification (e.g., City and Guilds, RSA/OCR, BTEC/Edexcel)	105	0.18	13	0.16
	NVQ at level 4 or equivalent	43	0.07	7	0.09
	At least one A level or equivalent	94	0.16	12	0.15
	At least one O level or equivalent	148	0.25	17	0.21
	No qualifications	45	0.08	10	0.13
Trade union membership	Yes	170	0.29	16	0.20
	No	420	0.71	64	0.80
Caring responsibility	Yes	172	0.29	21	0.26
	No	418	0.71	59	0.74

The survey was conducted in November 2014, after the Single Equalities Act 2010 implemented both the consolidation of discrimination regulations (including age) and the abolition of the Default Retirement Age which had allowed employers to force employees to retire at 65. The sample was collected using a market research firm and only people with an employment contract were contacted. Sampling stratification was used to guarantee a representative sample according to gender, industry and income.

### Measures

#### Job Satisfaction

Job satisfaction was measured using a six-item scale drawn from the European Social Survey (ESS, 2010). A sample item was: “My job makes me satisfied with what I have accomplished.” The responses were coded as follows: 1 = strongly disagree, 5 = strongly agree. The reliability coefficient using Cronbach’s alpha was 0.83.

#### Retirement Attitude

Retirement attitude refers to the positive or negative evaluation of retirement. In this study, it was measured with the following item, with a higher score referring to a positive evaluation: **“**Are you looking forward to full retirement.” The responses were coded as follows: 1 = Not at all, I am dreading it; 2 = Not really, I am apprehensive about it; 3 = I haven’t really thought about it; 4 – I’m relaxed about it; and 5 = Yes, I shall be pleased to retire/it will be a relief. This question was drawn from the Global Aging Studies survey (Leason, 2008).

#### Intended Retirement Age

Intended retirement age was captured by asking respondents to record the age at which they plan to retire. Respondents were given the following option of reporting: “I have no plans to retire.” These responses were excluded from the analysis.

#### Household Income

Respondents were asked to indicate their household weekly income before tax reduction. Response categories were divided into quintiles of the average weekly household income in the United Kingdom (Office for National Statistics, 2015). The modal category (32.5% of respondents) was the middle quintile indicating a weekly income of between £413 and £650 per week.

#### Controls

Given the extensive research identifying age, gender and health as known predictors of retirement intentions, these variables were included as controls. Age was measured as a numerical response to the question, ‘How old are you?’ Health was operationalized as a single item, ‘How is your health in general.’ The responses were coded as follows: 1 = very poor, 2 = rather poor, 3 = Moderate, 4 = Rather good, 5 = Very good. Gender was measured by means of one item differentiating between men (coded 1) and women (coded 2).

### Data Analysis

In this study, to test for the hypothesized relationships, contemporary practices of moderation and mediation advocated by Hayes (2013) were adopted. Based on multiple regression methods, a specialized form of moderated mediation, known as conditional process analysis modeling was used which examines and describes the conditional nature (that is, the moderating effect) by which a variable transmits its effect on another one (Hayes, 2013, p. 237). To estimate the conditional indirect effect of the independent variable job satisfaction (X), through the mediator retirement attitude (M), on the outcome variable intended retirement age (Y), with household income included as a moderator (W), the PROCESS macro for SPSS (v. 2.1.3.2) Model 59 was used (Hayes, 2013). This enabled the moderating effect of household income to be tested on all three paths simultaneously (as illustrated in **Figure 1**). In this analysis, age, health, and gender, were included as controls as these have all been found in previous studies to have a direct effect on intended retirement age (see for example Topa et al., 2009). The conditional process model generates (bias-corrected) 95% confidence intervals for the estimated indirect effects at various values of the moderator variable.

Conditional process analysis allowed the results to be probed at various point estimates by generating 5000 bootstrapped samples. Conditional indirect effects are calculated as the product of unstandardized regression weights for the path from the predictor to the mediator, and for the path from the mediator to the outcome variable. That is, the co-efficient for Path *a* × Path *b* were calculated separately for different levels of household income. In this analysis, they were calculated at three levels of household income: ‘high’ (mean plus one standard deviation), ‘mean’ household income (mean) and ‘low’ household income (mean minus one standard deviation).

## Results

In preliminary analyses, Average Variance Extracted (AVE), composite reliability and Cronbach’s alpha were used to test the independence of the variance for each of the model variables and were found to be satisfactory. Means, standard deviations, and bivariate correlations for the principal variables and controls are presented in **Table 2**. The correlation matrix suggests that there are indeed significant associations in the hypothesized direction between the model variables. The associations between job satisfaction and retirement attitude, on the one hand, appear to be unrelated to intended retirement age, which might indicate that indeed possibly moderators, like household income, are involved.

**Table 2:** Means, standard deviations and correlations between model variables (*n* = 590).

	Mean	*SD*	1	2	3	4	5	6
(1) Job satisfaction	3.87	*(0.69)*						
(2) Retirement attitude	3.72	*(1.18)*	-0.10^∗^					
(3) Intended retirement age	65.18	*4.39*	0.05	-0.28^∗∗^				
(4) Weekly household income	2.88	*1.11*	0.06	0.12^∗∗^	-0.20^∗∗^			
(5) Age	57.32	*4.81*	0.11^∗∗^	-0.15^∗∗^	0.33^∗∗^	-0.12^∗∗^		
(6) Health	2.21	*0.87*	0.17^∗∗^	0.00	0.03	0.10^∗^	0.03	
(7) Gender			0.05	-0.11^∗∗^	-0.08^∗^	-0.24^∗∗^	-0.08	-0.03

*^∗^denotes statistical significance at the 5% significance level. ^∗∗^denotes statistical significance at the 1% significance level.*

### Conditional Process Analysis

In **Table 3**, we present the results from the conditional process analysis. Using the PROCESS macro, in the first multiple regression, we tested whether household income (W) moderates the path from job satisfaction (X) to retirement attitude (Y) (depicted as path d in **Figure 1**). The outcomes indicated that job satisfaction did not have a significant negative association with retirement attitude (β = -0.02, CI: -0.04, 0.00). Importantly, the interaction term (computed as the product of household income and job satisfaction) appeared to have a significantly positive relationship to retirement attitude (β = 0.03, CI: 0.00, 0.05), controlling for age, health, and gender.

**Table 3:** The moderation effect of household income on retirement attitude.

	β	se	*t*	*p*	LLCI	ULCI
Constant	1.59	0.48	3.30	0.00	0.64	2.53
Job satisfaction	-0.02	0.01	-1.85	0.06	-0.04	0.00
Household Income	0.09	0.04	1.95	0.05	0.00	0.18
Interaction term (job satisfaction × household income)	0.03	0.01	2.43	0.02	0.00	0.05
Age	0.03	0.01	-3.29	0.00	-0.05	-0.01
Health	0.01	0.06	0.13	0.90	-0.10	0.12
Gender	-0.21	0.10	-2.16	0.03	-0.41	-0.02

*N = 590 Unstandardized coefficients are reported.*

In the second regression analysis, we tested whether household income (W) moderates the path from job satisfaction (X) to intended retirement age (Y) (depicted as path *e* in **Figure 1**). As shown in **Table 4**, job satisfaction did not have a significant direct effect on intended retirement age (β = 0.01, CI: -0.07, 0.09). However, the interaction between job satisfaction and household income appeared to be significantly positive (β = 0.08, CI: 0.01, 0.15). In the presence of the control variables, i.e., age, health, and gender, a significant association between retirement attitude and intended retirement age was found (β = -0.90, CI: -1.19, -0.62). It is notable that in the second regression analysis, respondent’s age and gender, were significantly associated with intended retirement age, respectively, for age: β = 0.24, CI: 0.17, 0.31; and for gender: β = -1.09, CI: -1.76, -0.42. The *R*^2^ for the second regression model was 0.21, indicating that 21% of the variance in intended retirement age could be accounted for by the model.

**Table 4:** The moderation effect of household income on the relationship between retirement attitude and intended retirement age.

	β	se	*t*	*p*	LLCI	ULCI
Constant	56.66	1.67	34.01	0.00	53.38	59.93
Retirement attitude	-0.90	0.14	-6.30	0.00	-1.19	-0.62
Job satisfaction	0.01	0.04	0.30	0.76	-0.07	0.09
Interaction term (retirement attitude × household income	0.01	0.13	0.05	0.96	-0.25	0.26
Household income	-0.67	0.15	-4.34	0.00	-0.98	-0.37
Interaction term (job satisfaction × household income)	0.08	0.04	2.12	0.03	0.01	0.15
Age	0.24	0.03	6.97	0.00	0.17	0.31
Health	0.16	0.19	0.82	0.41	-0.22	0.53
Gender	-1.09	0.34	-3.21	0.00	-1.76	-0.42

*N = 590 Unstandardized coefficients are reported.*

We hypothesized that different pathways would operate between job satisfaction, retirement attitude and intended retirement age, with varying levels of respondent’s household income. Probing the data at three levels of household income, **Table 5** shows, controlling for age, health, and gender, that there was no direct effect between job satisfaction and intended retirement age at any level of household income and so Hypothesis 1 was not supported with our data. However, fully supporting Hypothesis 2, conditional indirect effects were found between job satisfaction and intended retirement age, via retirement attitude for workers with a low- (β = 0.05, CI: 0.02, 0.09) and mean- (β = 0.02, CI: 0.01, 0.04) household income (minus one standard deviation and mean household income), but not for those with high household income (plus one standard deviation household income) (β = -0.01, CI: -0.03, 0.02).

**Table 5:** Conditional process analysis showing direct and indirect effects at three levels of household income.

	Direct effect Job satisfaction -> intended retirement age *N* = 590	{ Indirect effect 347 Job satisfaction -> retirement attitude -> intended retirement age (a × b) N = 590
Low household income (mean minus one standard deviation)	-0.07 (0.06)	**0.05** (0.02)
(Ci)	(-0.18, 0.04)	(0.02, 0.09)
Mean household income (mean)	0.01 (0.04)	**0.02** (0.01)
(Ci)	(-0.07, 0.09)	(0.01, 0.04)
High household income (mean plus one standard deviation)	0.10 (0.06)	-0.01 (0.01)
(Ci)	(-0.02, 0.21)	(-0.03, 0.02)

*CI = 95% confidence interval for indirect effect: if CI does not include zero, the indirect effect is considered statistically significant and is displayed in bold.*

**FIGURE 2:**
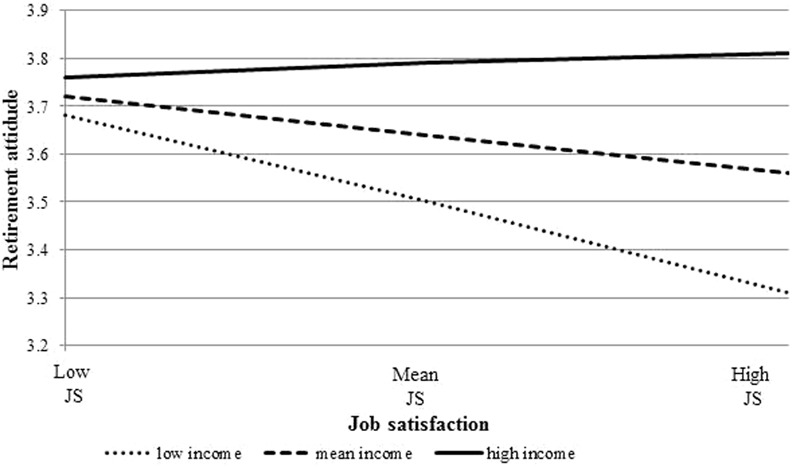
Interaction effect for job satisfaction and household income on retirement attitude.

The outcome of the interaction between job satisfaction and household income on retirement attitude is presented in **Figure 2** which illustrates that there is a significantly negative association between job satisfaction and retirement attitude for low-household income groups, but not for mean or high-household income group. The slopes’ graph illustrates that the negative effect of job satisfaction on retirement attitude was strongest for the low-household income category of workers.

## Discussion

### Reflection upon the Outcomes

As the working population ages (Shultz and Adams, 2007; Cridland, 2016) and a greater number of older employees remain in the labor force, understanding the dynamics of the retirement process (Shultz and Wang, 2011) and its relationship with work has assumed a renewed significance. Future workplaces will inevitably consist of older workers who face greater choice as well as greater uncertainty in relation to retirement timing. Therefore, understanding the dynamics of the retirement decision, including the influence of the impact of work-related attitudes on this, is an important theoretical and practical issue. Previous research into the impact of job satisfaction on intended retirement age has yielded contradictory results, suggesting that moderation and mediating effects may be relevant.

The goal of this study was to investigate further explanatory mechanisms in the relationship between job satisfaction and intended retirement age in a sample of 590 United Kingdom workers aged 50+. Drawing on theoretical frameworks which frame retirement decisions in terms of comparison between gains and losses (Anson et al., 1989; Newman et al., 2012; Chevalier et al., 2013), this empirical research examined whether job satisfaction exerts a direct effect on intended retirement age, and/or whether there are indirect effects with retirement attitude included as a possible mediator. In addition, we examined the possible moderating role of socio-economic status (household income) in the above-mentioned relationships.

With our outcomes, we found both some support for our hypothesized relationships and some unexpected results as well. First, we found that job satisfaction does not exert a direct effect on intended retirement age at any level of household income category and so Hypothesis 1 was not supported with our data. This finding is consistent with the insignificant results found in a number of other studies (McCune and Schmitt, 1981; Taylor and Shore, 1995; Adams and Beehr, 1998; Adams, 1999; Beehr et al., 2000; Davies and Cartwright, 2011; Smith et al., 2011; Post et al., 2013). It seems that we may cautiously conclude that a higher household income does not relax the individual’s complex decision-making process around intended retirement age. Possibly, the decision to retire is related to a broader concept than the job itself such as the meaning of work for the individual employee. That is to say, for most people work is more than securing income; work is a prominent element in one’s life that provides highly valued psychological and social aspects (Chalofsky, 2003; Cartwright and Holmes, 2006; Fasbender et al., 2016). It might be that once one’s basic needs are fulfilled, which applies to many people in the Western world, meaning of work becomes an even stronger factor in comparison with employees from less developed countries.

As regards Hypothesis 2, our results confirmed the mediating role of retirement attitude in the relationship between job satisfaction and intended retirement age at specific levels of socio-economic status: the mediation effect was found for low- and mean-level household income individuals, yet not for the high-income group (thereby fully supporting Hypothesis 2).

These findings reveal a critical insight into the role of retirement attitude in the light of intended retirement age, and also shed more light on the mechanism through which job satisfaction influences intended retirement age. For workers from all three categories of socio-economic status, a significant main effect of retirement attitude on intended retirement age was found: older workers who positively look forward to retirement report an earlier intended retirement age. These findings are consistent with others studies which have examined the role of retirement attitude on intended behavior (Zappala et al., 2008; Davies and Cartwright, 2011; Cochran et al., 2012).

By examining retirement attitude as a possible mediator between job satisfaction and intended retirement age, we have been able to reveal greater depth of insight into the underlying relationships. Although job satisfaction appears not to have any direct effect on intended retirement age, by investigating the moderating effects of socio-economic status, we show that job satisfaction does in fact exert an indirect effect on intended retirement age for specific categories of older workers by modifying their assessment of retirement attitude. The slope analysis shows that when job satisfaction was low, all socio-economic groups held a broadly positive attitude to retirement, in turn, leading to earlier intended retirement age. However, at mean-and high-levels of job satisfaction, different patterns were observed between socio-economic groups. In the high household income group, there was no relationship between level of job satisfaction and retirement attitude. However, for mean-and low-household income older workers, lower levels of job satisfaction are associated with progressively poorer evaluations of retirement. This suggests that a highly satisfied/low household income older worker will hold a negative evaluation of retirement. It is likely that for such a person, retirement would entail the loss of the job as a rewarding and fulfilling life sphere that might not be substituted easily by other retirement benefits, such as satisfactory retirement income, future positive social/leisure experiences in retirement. It therefore represents a significant life loss. By contrast, a high household income older worker’s evaluation of retirement appears to be unaltered by the level of job satisfaction, be it higher or lower. For higher household income older workers, retirement attitude is likely to be determined by a range of factors such as social status, expected access to leisure resources/activities, and personal relationships arising out of enhanced social capital, and may operate largely independently of their immediate feelings about the job. In addition, as indicated above, it might be that the meaning work has for an individual is a key factor in the decision-making process about retirement, over and above the fulfillment of basic needs such as salary provision or immediate characteristics of the job.

These findings offer important theoretical contributions to the scholarly literature in this field. Previously, researchers have proposed models that conceptualize retirement attitude as a ‘balanced’ outcome, and a careful evaluation of the respective gains and losses associated with the ending of work and the onset of retirement (Anson et al., 1989; Newman et al., 2012). Our data are consistent with a comparative approach and indicate that job satisfaction does indeed appear to influence a generalized retirement attitude, but only for workers with mean- and low-household incomes. For high household income workers, other factors, such as meaning of work, social status, and relationships stemming from being in employment may compensate or substitute for any loss of higher job satisfaction, and so job satisfaction in itself will have relatively less impact on retirement attitude. This line of reasoning implies that high household income individuals undergo a more complex decision-making process when comparing the pros and cons of the relative merits of the satisfaction they gain from their specific job alongside the other substantial gains and losses in retirement. For lower household income workers with low job satisfaction [arising possibly from work which is physically or psychologically unpleasant, and more often, an immediate danger for their sustainable employability, see Van der Heijden and De Vos (2015)], retirement is likely to be evaluated as a substantive ‘gain,’ and so be relatively more attractive, because it is expected to help terminate an undesirable life activity. On the contrary, the finishing of a highly satisfying job in the context of lower household income is likely to be evaluated negatively.

Our outcomes regarding the moderating effect of household income are in line with the argumentation following from the comparison approach to attitudes to retirement (Newman et al., 2012) but only for lower socio-economic status workers. The findings in this study are also consistent with those of Post et al. (2013), who reinforced the importance of financial concerns in influencing retirement intentions, and highlight the importance of context in understanding of socio-economic status in the dynamics of the relationship between work and retirement (Hennekam and Herrbach, 2013).

### Limitations and Recommendations for Future Research

The present study has limitations. Firstly, all data have been collected using questionnaires (through telephone interviews), and by using self-reported data only, opening up the possibility of response set consistencies and common-method bias (Podsakoff et al., 2003) and potential effects where responses to one question cognitively cue another. Secondly, all data have been collected at one point in time, that is, the study is cross-sectional. As noted earlier, the cross-sectional designed required focus on intended retirement age instead of actual retirement age. These issues imply that further research, preferably using multi-rater designs (for instance combining employee and supervisor and/or partner ratings) is needed in order to address the issues of causality and research on actual retirement behaviors. Research using multi-wave designs can provide more specific information about the stability and change of the variables, and about cross-lagged (i.e., over time) relationships than our cross-sectional approach (Taris and Kompier, 2003; De Lange, 2005). Although we captured *intended* retirement age and not one’s *actual* retirement age, previous scholars have robustly defended the use of intended retirement age as viable sources of information about retirement decisions (Prothero and Beach, 1984; Beehr and Bennett, 2007; Solem et al., 2016). Therefore, we believe that our results are noteworthy and provide good challenges for future research and cross-validation.

Given the current cross-sectional methodology, we cannot of course exclude other explanations for our outcomes. For instance, one possible alternative that forms a good basis for future empirical approaches is that the assumed direction of causality is reversed: the broader attitude to retirement itself might influence an older worker’s job satisfaction. However, in our opinion, this appears to be a less probable explanation given that chronologically work precedes retirement, thereby suggesting that attitudes to work precede attitudes to retirement. A further alternative possible explanation, worthy of more explicit future investigation, is that a person’s disposition or personality (Newman et al., 2012) might influence these relationships as well. For instance, individuals with higher core self-evaluations may have greater belief in their ability to adjust to retirement than those with lower self-efficacy (Topa and Alcover, 2015; Valero and Topa, 2015). Likewise, individuals predisposed to general satisfaction may expect satisfaction across both job and retirement roles, whereas individuals predisposed to general dissatisfaction are assumed to perceive dissatisfaction across different life spheres (Schmitt and Pulakos, 1985).

Thirdly, further research is needed to investigate the robustness of our findings, and to determine the extent to which our findings generalize to other occupational settings and/or to other countries (Fouad and Arbona, 1994). Fourthly, following up on the reflections given above, we might investigate empirical models wherein the possible influence of factors such as sense-making and meaning of work for the individual in predicting intended retirement age are incorporated as well.

Another possible moderator might be age-related stereotyping, suggesting that the relationship between the model variables might be influenced in case the employee suffers from negative attitudes from important key figures, such as one’s direct supervisor, at a later age (Van der Heijden et al., 2009; Karpinska et al., 2013). Future research is needed to empirically investigate the credibility of these lines of reasoning. Moreover, it might be interesting to use the Job Demands-Resources (JD-R) model (Bakker and Demerouti, 2007) that has proven to be applicable to many occupational and organizational settings as a guiding framework in future research on retirement decisions.

### Practical Implications

Our findings have important implications for practice, both for employers and employees. In the context of an aging workforce, and the current, highly prevalent imperative on older workers to extend their working life and to delay retirement, it is important to be aware of the complex nature of the interaction between one’s job, retirement evaluations and socio-economic status. Older workers with a higher socio-economic status are able to directly respond to a lack of job satisfaction, by means of earlier retirement, however, it seems that in many occasions they do not do so by means of considering early retirement. We believe that this might be due to the many other aspects that work may provide, such as sense-making and meaning in life, social networks, and structure, to mention but a few.

Both quantitative and qualitative previous studies highlight the relationship between agency and income/wealth in later life (McNair et al., 2004; Flynn, 2010). In fact, there is a view that government and employers should focus public and HR policies, respectively, on low income workers so as to enhance their agency and to give them more choice over when and how to retire (Lain, 2012).

From the perspective of the levers for action available to employers, it follows that actions taken to increase job satisfaction should be the main focus of attention (see Alegre et al., 2016 for an overview of the antecedents of job satisfaction). However, our outcomes also highlight a potential dilemma for increasing the job satisfaction for lower household older workers. In particular, increases in job satisfaction for lower household income workers are likely to make the prospect of retirement less attractive, and therefore utmost care must be taken around the circumstances and conditions under which lower income employees will continue their work when getting older. Both direct supervisors and HR managers are very important in this regard as they are key figures in protecting and enhancing workers sustainable employability throughout their career (Van der Heijden and De Vos, 2015).

## Ethics Statement

This study was carried out in accordance with the recommendations of Middlesex University Business School Ethics Committee with written informed consent from all subjects. All subjects gave written informed consent in accordance with the Declaration of Helsinki. The protocol was approved by Middlesex University Business School. All participation was voluntary. No participants were minor or vulnerable people.

## Author Contributions

MF performed the data collection. ED and BH were responsible for the study design. ED performed the data analysis. ED and BH were responsible drafting of the manuscript. BH, ED, and MF made critical revisions to the paper for important intellectual content. ED and BH provided statistical expertise. MF obtained funding.

## Conflict of Interest Statement

The authors declare that the research was conducted in the absence of any commercial or financial relationships that could be construed as a potential conflict of interest.
